# Draft genome of the living fossil *Ginkgo biloba*

**DOI:** 10.1186/s13742-016-0154-1

**Published:** 2016-11-21

**Authors:** Rui Guan, Yunpeng Zhao, He Zhang, Guangyi Fan, Xin Liu, Wenbin Zhou, Chengcheng Shi, Jiahao Wang, Weiqing Liu, Xinming Liang, Yuanyuan Fu, Kailong Ma, Lijun Zhao, Fumin Zhang, Zuhong Lu, Simon Ming-Yuen Lee, Xun Xu, Jian Wang, Huanming Yang, Chengxin Fu, Song Ge, Wenbin Chen

**Affiliations:** 1 BGI-Shenzhen, Shenzhen, 518083 China; 2 The Key Laboratory of Conservation Biology for Endangered Wildlife of the Ministry of Education, College of Life Sciences, Zhejiang University, Hangzhou, 310058 China; 3 State Key Laboratory of Systematic and Evolutionary Botany, Institute of Botany, Chinese Academy of Sciences, Beijing, 100093 China; 4 BGI-Qingdao, Qingdao, 266555 China; 5 State Key Laboratory of Bioelectronics, School of Biological Science and Medical Engineering, Southeast University, Nanjing, 210096 China; 6 Laboratory of Systematic & Evolutionary Botany and Biodiversity, Institute of Ecology and Conservation Center for Gene Resources of Endangered Wildlife, Zhejiang University, Hangzhou, 310058 China; 7 State Key Laboratory of Quality Research in Chinese Medicine and Institute of Chinese Medical Sciences, Macao, China; 8 Stanley Ho Centre for Emerging Infectious Diseases, Faculty of Medicine, The Chinese University of Hong Kong, Shatin, Hong Kong; 9 BGI-Wuhan, BGI-Shenzhen, Wuhan, 430074 China; 10 James D. Watson Institute of Genome Sciences, Hangzhou, 310058 China

**Keywords:** Ginkgo genome, Evolution of LTR-RTs, Tandem gene duplication, Plant defense mechanism, Gymnosperm evolution, Whole genome duplication

## Abstract

**Background:**

*Ginkgo biloba* L. (Ginkgoaceae) is one of the most distinctive plants. It possesses a suite of fascinating characteristics including a large genome, outstanding resistance/tolerance to abiotic and biotic stresses, and dioecious reproduction, making it an ideal model species for biological studies. However, the lack of a high-quality genome sequence has been an impediment to our understanding of its biology and evolution.

**Findings:**

The 10.61 Gb genome sequence containing 41,840 annotated genes was assembled in the present study. Repetitive sequences account for 76.58% of the assembled sequence, and long terminal repeat retrotransposons (LTR-RTs) are particularly prevalent. The diversity and abundance of LTR-RTs is due to their gradual accumulation and a remarkable amplification between 16 and 24 million years ago, and they contribute to the long introns and large genome. Whole genome duplication (WGD) may have occurred twice, with an ancient WGD consistent with that shown to occur in other seed plants, and a more recent event specific to ginkgo. Abundant gene clusters from tandem duplication were also evident, and enrichment of expanded gene families indicates a remarkable array of chemical and antibacterial defense pathways.

**Conclusions:**

The ginkgo genome consists mainly of LTR-RTs resulting from ancient gradual accumulation and two WGD events. The multiple defense mechanisms underlying the characteristic resilience of ginkgo are fostered by a remarkable enrichment in ancient duplicated and ginkgo-specific gene clusters. The present study sheds light on sequencing large genomes, and opens an avenue for further genetic and evolutionary research.

**Electronic supplementary material:**

The online version of this article (doi:10.1186/s13742-016-0154-1) contains supplementary material, which is available to authorized users.

## Background

*Ginkgo biloba* L. is one of the best-known and most distinctive trees, and represents one of the four extant gymnosperm lineages (cycads, ginkgo, conifers and gnetophytes) and has no living relatives. Cycads are widely accepted to be the most closely related lineage [1–4] but the debate continues [5].

Gingko is a living fossil that has remained essentially unchanged in terms of gross morphology for more than 200 million years [6, 7]. It has survived glaciations as a relic in China [8, 9] followed by a human-aided global redistribution [10], and thus provides an inspiring example of how humans can help a species survive and renew. The infamous resilience of ginkgo has enabled it to become widespread and popular across the world. This resilience includes an outstanding resistance or tolerance to both herbivores and pathogens, accounting in part for the longevity of individual trees, and also, in turn, for the longevity of the species [11]. At least three separate defense systems act in ginkgo in response to herbivore attacks: i) repellence and antifeedance due to the presence of flavonoids and terpenic trilactones such as ginkgolides and bilobalides [12]; ii) activation of direct defense genes and the production of plant secondary metabolites including glycosylated flavonoids; iii) emission of specific volatile organic compounds (VOCs) mainly built from terpenes (e.g., sesquiterpenes α-copaene and β-caryophyllene) that potentially activate indirect defenses by attracting predators of browsing insects [13]. Ginkgolides and bilobalides have been linked to resistance to fungi [12] and possibly endophytic bacteria [14], although the mechanism of resistance to bacterial pathogens remains unclear.

Although numerous studies have focused on the aforementioned unique features of this fascinating species, and despite the increasing availability of genome-derived resources for ginkgo [15–18], our comprehensive and in-depth understanding has been impeded by the lack of a well-resolved and fully annotated genome that would also facilitate studies on the evolution of land plants, in particular gymnosperms. Furthermore, a complete ginkgo genome will facilitate the assembly and annotation of the published genome drafts of *Picea abies* [19], *Picea glauca* [20, 21] and *Pinus taeda* [22–26], along with other newly-reported gymnosperm genomes, as well as the transcriptomes of *Cycas revoluta*, *Ephedra trifurca* and *Pinus canariensis* [27]. Despite the rapid advance in the publication of complete genome sequences for diverse plant species, the belated publication of the ginkgo genome may in part result from its large size, which was estimated to be 11.75 Gb [28]. The vast amount of repeated sequences in gymnosperm genomes, as illustrated in the genome draft of *P. abies* [19], further hampers ambitions to address this issue.

In this study, we present the ginkgo genome sequence based on the high-quality assembly, annotation and analysis of genomic structures and evolution, and provide new insight into the evolution of large genomes and multiple defense mechanisms.

## Data description

Genomic DNA was extracted from endosperm tissue from ginkgo seeds that develop directly from female gametophytes without fertilization and thus contain haploid genomes without undergoing genetic recombination. All paired-end libraries and one mate-pair library (2 kb) were constructed using DNA extracted from a single seed. The additional five mate-pair libraries (2 − 40 Kb) were constructed for scaffolding using DNA from other seeds from the same sampled tree (see details in Additional file 1: Table S1). These eight libraries were used only for scaffolding procedures to avoid the possible introduction of heterozygosity. Data were generated using a Hiseq 2000/4000 platform from 1253.09 Gb clean data (Additional file 1: Table S2). RNA was isolated from female and male reproductive organs and a 2-year seedling, respectively. Preparation of the cDNA library and sequencing was performed as described previously [29]. A total of 6.30 Gb, 6.40 Gb and 6.40 Gb of raw data were obtained from female, male and seedling samples, respectively, using an Illumina Hiseq 2000 (Additional file 1: Table S3). Further details about sample collection, DNA/RNA extraction, library construction and sequencing can be found in the Methods section. All genome data have been uploaded to *Giga*DB [30].

## Analyses

### Genome sequencing and assembly

Whole genome sequencing of ginkgo yielded 189.84× coverage raw sequence data. After filtering, 120.79× high-quality reads, comprising ~100× from paired-end libraries and ~20× from mate-pair libraries, remained for genome assembly (Additional file 1: Table S2). The ginkgo genome was estimated to be 10.00 Gb in size (Additional file 1: Table S4) with a high proportion of repeat elements (Additional file 2: Figure S1). All clean data were used to generate a draft genome assembly, followed by gap filling. The assembled draft genome is 10.61 Gb in length (including 493 Mb N’s) with N50 values of 48.2 kb for contigs and 1.36 Mb for scaffolds, respectively (Table 1).

**Table 1:** Assembly and annotation of the ginkgo genome

Category		Number	N50 (bp)	Size (bp)	Percentage of the assembly (%)
Contigs		6,990,752	48,207	10,115,209,138	--
Scaffolds		6,459,773	1,358,237	10,608,657,252	100.00
Repetitive sequence		--	--	8,124,064,469	76.58
Transposable elements	LTR	--	--	6,434,519,114	60.65
	DNA	--	--	354,935,994	3.34
	LINE	--	--	460,463,526	4.34
	SINE	--	--	261,728	0.00
	Unknown	--	--	2,694,184,164	25.39
Annotated genes		41,840	--	26,829/279/7884^a^	10.58

^a^Average mRNA length, exon length and intron length, respectively

To evaluate the sequencing randomness and assembly quality, a fraction of reads (insert size of 500 bp and sequencing depth of 10.5-fold, Additional file 1: Table S2) was mapped to the genic region of the assembled genome, and the sequencing depth for each base exhibited a Poisson-like distribution (Additional file 2: Figure S2), suggesting no obvious bias for sequencing and assembly. Subsequent alignment of 17,902 expressed sequence tags (ESTs) to the assembled genome, along with 42,243 and 32,088 transcripts assembled from RNA-seq data from ginkgo seeds and leaves, resulted in 94.45, 92.68 and 92.57% being properly mapped, respectively (identity cutoff = 0.90, coverage ratio cutoff = 0.90).

### Genome annotation

Using a combination of *de novo* and homology-based methods, up to 76.58% of the assembled sequences were found to be repeated sequences comprising transposable elements and tandem repeats, of which 79.20% were of the long terminal repeat (LTR) type (Additional file 1: Table S5 and Additional file 2: Figure S3; see further details below). Application of the k-mer frequency-based approach [31] resulted in an even higher proportion of repeat content (~84.43%), highlighting the challenges involved in assembling the repetitive elements in the ginkgo genome from limited short-read sequencing data.

Gene models were predicted using protein sequences from five land plants (*Selaginella moellendorffii*, *Picea abies*, *Pinus taeda*, *Arabidopsis thaliana* and *Oryza sativa*) coupled with transcriptomes assembled from RNA-seq data and EST data downloaded from NCBI (Additional file 1: Table S6). The length distributions of four categories of annotated genes (gene, coding sequence (CDS), exon and intron) were compared for the five species (Additional file 2: Figure S4). Calculation if the completeness of gene sets using BUSCO [32] (V1.1b) resulted in the identification of 707 complete single-copy BUSCOs (73.95% of the 956 known BUSCO groups), of which 308 were duplicated. Function annotation via mapping to functional databases identified 68.12% protein sequences with known homologous genes (Additional file 1: Table S7). The presence of 31.88% of proteins with no known function indicates a large proportion of highly diverged, species-specific genes in the ginkgo genome.

**Fig. 1:**
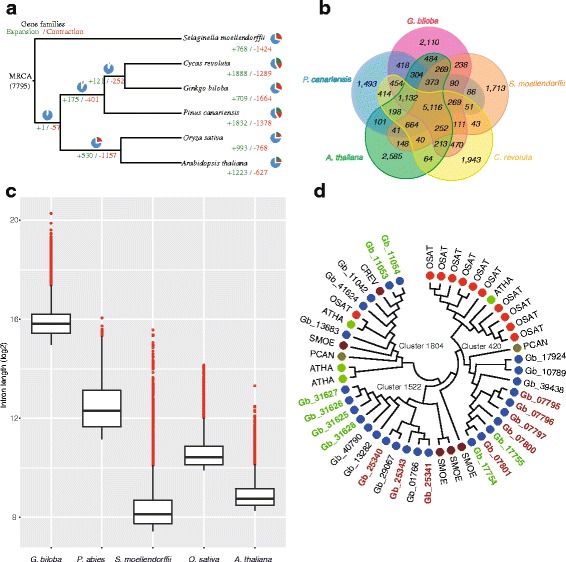
Phylogenetic relationships and comparative genomic analyses. **a** Phylogenetic relationships and number of gene families displaying expansion and contraction. **b** Comparison of the number of gene families in the five land plants *Ginkgo biloba*, *Cycacs revoluta*, *Pinus canariensis*, *Selaginella moellendorffii* and *Arabidopsis thaliana*. **c** Comparison of the longest 10% of introns in the five land plants. **d** Phylogenetic tree of three orthologous gene families indicating gene duplication and tandem distribution. The colors of solid circles represent species. Gene IDs of ginkgo start with ‘Gb’, and red and green text represents tandem distribution in different scaffolds. CREV, *C. revoluta*, PCAN, *P. canariensis*, SMOE, *S. moellendorffii*, OSAT, *O. sativa*, ATHA, *A. thaliana*

Of the 41,840 predicted ginkgo genes, 30,209 were assigned with high confidence based on supporting expression data, which is a slightly higher proportion than *P. abies* (28,354) [19] and far more than *P. taeda* (15,653; Additional file 1: Table S8) [23, 24]. The average length of *G. biloba* mRNA or CDS sequences was longer than that of *P. abies* (Additional file 2: Figure S4). Following comparison with the genomes of *S. moellendorffii*, *P. canariensis, Cycas revoluta*, *A. thaliana* and *O. sativa* (Additional file 1: Table S9), ginkgo genes were clustered into 12,303 families (Additional file 2: Figure S5). Phylogenetic reconstruction based on 920 single copy orthologous genes indicates that ginkgo is more closely related to cycads than is *P. canariensis* (Fig. 1a), further supporting the prevailing hypothesis of gymnosperm phylogeny [1–4].

We identified 2110 gene families unique to ginkgo, and 5116 orthologous gene families shared by the five land plants were analyzed (Fig. 1b). The ginkgo-specific gene families (including 11,105 genes, of which 4247 were functionally annotated in the KEGG database) are remarkably enriched in eight specific pathways/functions, including ATP-binding cassette (ABC) transporters, monoterpenoid biosynthesis and phenylpropanoid biosynthesis (Additional file 1: Table S10). Signals characteristic of expansion and contraction were detected in 709 and 1664 gene families, respectively (Fig. 1a). GO enrichment analysis of a subset of 223 gene families of significantly altered size (*P* < 0.05) revealed that pathways associated with the response to biotic stimuli, such as the defense response (Cluster 368: 21 genes in ginkgo vs. 1 gene in *S. moellendorffii*, *O. sativa* and *P. canariensis*) and plant-type cell wall organization (16 genes encoding malate dehydrogenase in both Cluster 719 and Cluster 1793) were particularly enriched. Highly represented molecular functions included protein kinase activity (*FLS2* and *EFR* gene families in Cluster 38 that have a key role in plant-pathogen interactions), transferase activity, transferring hexosyl groups (involved in conjugation of the growth hormone indole-3-acetic acid [33] and glucosinolate biosynthesis), and terpene synthase activity (functioning in the defense system mainly via alpha-bisabolene synthase [34].

### Evolution of LTR-RTs

The ginkgo genome is mainly composed of repeated sequences, mostly LTR-RTs (58.34% of assembled sequences), of which two superfamilies, *Ty3/Gypsy* and *Ty1/Copia*, account for 45.63 and 12.71% of all assembled sequences, respectively. Two rounds of BLAST similarity searches identified 24,090 vs. 17,564, 28,352 vs. 10,915, 2416 vs. 1790, 728 vs. 2727, and 365 vs. 494 *Ty1/Copia* and *Ty3/Gypsy* domains in the genomes of *G. biloba*, *Zea mays*, *P. abies*, *Physcomitrella patens* and *Populus trichocarpa*, respectively.

**Fig. 2:**
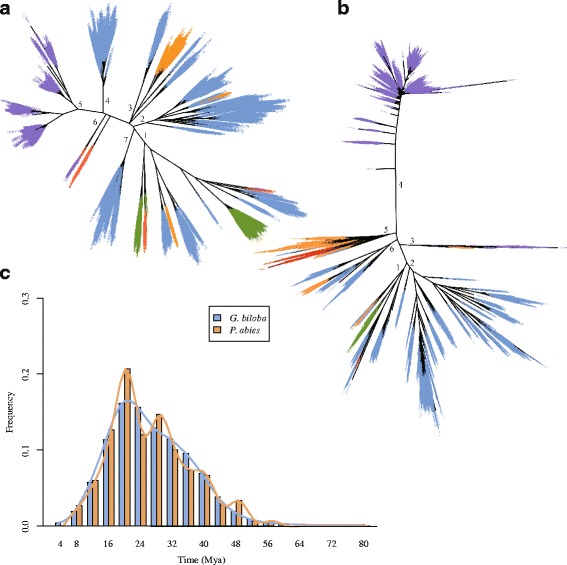
Evolution of LTR-RTs in ginkgo. **a** Phylogenetic relationships within the *Ty3/Gypsy* superfamily in the five land plants. Blue: *Ginkgo biloba*; green, *Physcomitrella patens* (moss); orange, *Picea abies*; red, *Populus trichocarpa*; purple, *Zea mays*. **b** Phylogenetic relationships within the *Ty1/Copia* superfamily in the five land plants. **c** Comparison of the timing of LTR-RTs insertions between *G. biloba* and *P. abies*

To investigate the evolution of transposable elements (TEs) in land plants, phylogenetic trees of domains in reverse transcriptase genes were constructed for both *Ty1/Copia* and *Ty3/Gypsy* elements. In the phylogenetic tree of the *Ty3/Gypsy* superfamily, LTR-RTs from ginkgo were clustered in five (1, 2, 3, 4 and 7) of the seven major clades. Clade 1 was composed of elements from all five species (Fig. 2a), indicating conservation among land plants, and the origins of the other clades postdating species divergence. Notable ginkgo-specific expansions were observed in clades 4 and 7, representing two subfamilies, and in clade 2, which included a minority of components from *P. abies*. Despite a closer phylogenetic relationship and shared clades 2 and 3, ginkgo displayed substantially higher diversity and abundance within the *Ty3/Gypsy* superfamily than the other analyzed gymnosperm (*P. abies*), possibly indicating ancient origins for these diverse clades (subfamilies) followed by a gradual and/or rapid diversification. The two clades revealed for *Z. mays* (5 and 6) both diverged more recently than the other major clades, and while clade 6 shared by maize and poplar was narrow, clade 5, which is specific to maize, was far more diverse and abundant, indicating within-clade divergence intermediately followed by recent expansion of the *Ty3/Gypsy* family in maize.

The *Ty1/Copia* superfamily exhibited a slightly different pattern (Fig. 2b), with four of the six major clades (1, 2, 5 and 6) present in ginkgo. Clade 1, shared by all five land plants, is presumably the most conserved. All four clades were less scattered in the phylogenetic tree, and clustered more closely to clade 1, than their counterparts in the *Ty3/Gypsy* tree, consistent with a more conserved evolution, as described previously [35–38]. Clade 2, the sole ginkgo-specific clade, was both highly diverse and abundant, and was a sister clade to clade 1, the most conserved clade, indicating an earlier split from the ancestral gene than occurred in the *Ty3/Gypsy* superfamily. Relative to clade 2, clade 6 is less well conserved in ginkgo, and clade 5 even less so. Even so, this superfamily appears to be more diverse and abundant in ginkgo than in *P. abies*, which was expanded mainly in clade 5. The majority of elements from maize similarly clustered in the species-specific clade 4, connected by a long branch, while a few members were clustered with spruce-derived elements in the narrow clade 3.

Furthermore, to estimate the activity of LTR-RTs at the molecular level, 47,342 putative complete LTR-RTs were identified in the ginkgo genome, and the insertion time for each pair of LTR-RTs [39] had a mutation rate of 2.2 × 10^-9^ substitutions per base per year [19]. This result suggests that the amplification of LTR-RTs occurred largely between 16 and 24 million years ago (mya; Fig. 2c), consistent with the timescale proposed for *P. abies* [19].

### TE insertions in introns

A comparison of gene models for the five land plants revealed that the average length of the longest 10% of introns (13,607 for *G. biloba*, 5069 for *P. abies*, 10,034 for *S. moellendorffii*, 12,152 for *O. sativa* and 11,275 for *A. thaliana*) in ginkgo was significantly longer (*P* <2.0 × 10^-6^, Welch Two Sample *t*-test) than in the other four land plants (Fig. 1c). Similar results were also reported previously for *P. glauca* and *P. taeda* [21, 24, 40]. Repeat elements occurred in 26.56% of introns in ginkgo, whereas they accounted for 65.48% of the total sequence length of introns. In contrast, repetitive elements only accounted for 2.94% of intron sequences in *P. glauca*, even though these elements occur in 32.4% of genes [40]. Furthermore, comparison of gene structures (Additional file 2: Figure S6) as well as average intron length and genome size (Additional file 2: Figure S7) revealed that the length and number of exons in ginkgo are conserved, while introns vary substantially, consistent with these elements in conifers [40].

Further comparison of the distribution of repeats revealed a high percentage of repeats in both introns and intergenic regions, reaching a maximum of 69%. For regions with a repeat ratio less than 44%, the frequency of introns was higher than that of intergenic regions. Conversely, for regions with a repeat ratio of 44% or greater, the frequency of repeats in introns was lower than intergenic regions (Additional file 2: Figure S8). Transposon insertions tend to accumulate in genes that are expressed at lower levels [41], which may be driven by negative selection due to increased transcriptional cost for longer transcripts [42].

### Gene duplications

**Fig. 3:**
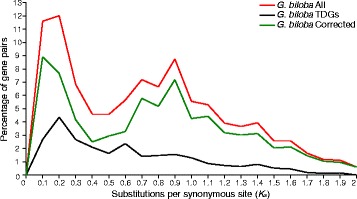
*K*_s_ distribution of clustered genes in ginkgo. *K*_s_ distributions were plotted for three sets of genes; all clustered genes (*red*), clusters of tandem duplicate genes (TDGs; *black*) and clustered genes excluding TDGs (*green*)

Whole genome duplication (WGD) and tandem duplication resulting from unequal crossover represent two dominant mechanisms for generating gene copies. WGDs are usually identified from the *K*_s_ (a measure of the substitutions per synonymous site) distribution of paralogs, or from gene collinearity data. We identified as few as 40 syntenic gene pairs within the entire gene set of the ginkgo genome. Thus, calculation of the *K*_s_ value of paralogs of gingko [43] was based on the gene cluster results of OrthoMCL (release 5) [44]. WGD may lead to peaks of gene duplication, whereas accumulation of tandem duplicate genes (TDGs) may affect the distribution of *K*_s_ values. Two peaks were observed in the corrected *K*_s_ distribution (after removing TDGs) of gingko (i.e., *K*_s_ = 0.1 − 0.2 (peak 1) and *K*_s_ = 0.7 − 1.0 (peak 2; Fig. 3). The timing of the two WGDs at peaks 1 and 2, estimated from the mutation rates (per base per year) of 0.68 × 10^-9^ [45], was found to be between 74 and 147 mya, and between 515 and 735 mya, respectively. The latter of these time periods is consistent with that previously reported for seed plants [46–48], while the former time period is far later than when *Gingko* and conifers diverged, indicating a potential independent WGD event postdating the origin of *Ginkgo* by at least 170 mya [49].

We detected 2061 gene clusters containing 5201 TDGs (Additional file 1: Table S11) and an unambiguous peak in the *K*_s_ distribution of these TDGs in ginkgo that corresponded to the predicted recent WGD (Fig. 3). Two sets of TDGs (Gb_25430, Gb_25431 and Gb_25343 on scaffold4299, and Gb_31625, Gb_31626, Gb_31627 and Gb_31628 on scaffold5904; the clade in Cluster 1522 in Fig. 1d) were identified that belong to the same shikimate O-hydroxycinnamoyl transferase family, which forms a kernel in the biosynthesis of plant secondary metabolites including stilbenoid, diarylheptanoid and gingerol. A tandem distribution was also observed for gene families related to the gibberellin (GA) receptor GID1 (Fig. 1d, the clade in Cluster 420) involved in transducing GA signaling and inducing a wide range of plant growth responses. Cluster 1804, comprising Gb_11042, Gb_11053, Gb_11054, Gb_13683 and Gb_41624 encoding pathogenesis-related protein 1 that functions in plant hormone signal transduction and plant-pathogen interactions, was highly diverged between ginkgo and other land plants, and also within ginkgo (Fig. 1d).

### Resistant genes

**Fig. 4:**
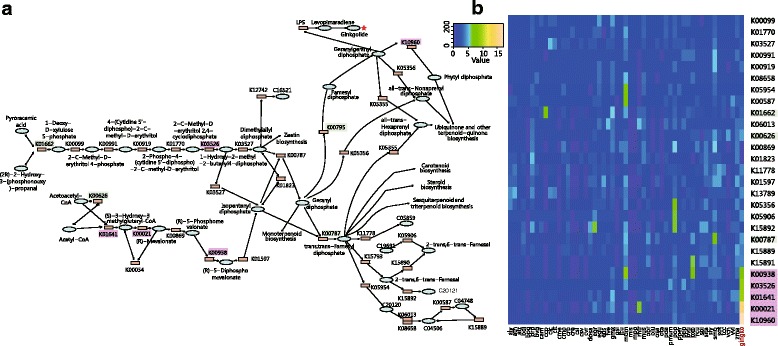
Gene expansion of terpenoid backbone biosynthesis in ginkgo. **a** Backbone pathways of terpenoid biosynthesis and enriched genes in ginkgo. Genes names shaded in green apparently underwent expansion in ginkgo based on comparison with *S. moellendorffii*, *P. canariensis, C. revoluta*, *A. thaliana* and *O. sativa.* Gene names shaded in purple apparently underwent expansion in ginkgo based on comparison with all released plant genomes in the KEGG database. Expansion in both K03526 and K01641 gene families was suggested by both comparisons. **b** The number of each gene in the pathways of all released plant genomes in the KEGG database. Genes in purple and green shadow correspond to expansions as described in (**a**). Full names for species can be found at http://www.genome.jp/kegg/catalog/org_list.html

Considerable expansion was also uncovered for diverse gene families related to multiple defense mechanisms in ginkgo. The glucosinolate biosynthesis gene family comprises 29 genes involved in core pathways of both pungent components and flavonoids (flavone and flavonol) biosynthesis. The α-bisabolene synthase gene family, absent in both *A. thaliana* and *O. sativa*, is composed of 28 genes participating in terpene synthase activity, carbon-oxygen lyase activity and magnesium ion binding. Meanwhile, we observed a high incidence of gene duplication in an array of genes in two pathways of defensive metabolites (Fig. 4a), one involving the six ginkgolide/bilobalide biosynthesis genes *DXS* (1-deoxy-D-xylulose-5-phosphate synthase, K01662), *HDS* (4-hydroxy-3-methylbut-2-en-1-yl diphosphate synthase, K03562), *GPPS* (geranyl-diphosphate synthase, K00787), *FPPS* (farnesyl-diphosphate synthase, K00795), *HMGS* (HMG-CoA synthase, K01641), and *AACT* (acetyl-CoA acetyl- transferase, K00626) [16]. We also compared the copy number of each gene in this pathway between ginkgo and all other species in the KEGG database (release-76), and observed a remarkable expansion in K00938, K003526, K01641, K00021 and K10960 (Fig. 4b, shadowed in purple) in ginkgo. The other high incidence of gene duplication occurred in four sets of genes involved in flavonoid biosynthesis (ko00360 in KEGG) comprising *CHS* (chalcone synthase), *F3’H* (flavonoid 3′-hydroxylase), *FLS* (flavonol synthase) and *DFR* (dihydromyricetin reductase).

The most remarkable duplication was detected in a gene family involved in plant-pathogen interactions and related to leucine rich repeat (LRR) receptor-like serine/threonine-protein kinase flagellin-sensitive 2 (*FLS2*) and EF-Tu receptor (*EFR*). *FLS2*-coded pattern recognition receptors (*PRR*s) recognize the highly conserved N-terminal epitope (*flg22*) that is a feature of the main bacterial flagella protein flagellin [50]. The *EFR*-coded *PRR*s for bacterial *EF-Tu* function similarly [51]. In gingko, the family that includes both *FLS2* and *EFR* contains as many as 62 genes, far more than that in *A. thaliana* (one copy of *FLS2* and one copy of *EFR*). Thus, *FLS2* could be considered an important element of the immune system in ginkgo. Furthermore, five additional duplicate genes (*RIN4, HSP90, CEBiP, CaMCML* and *PR1*) were also enriched in this pathway (map04626 in KEGG).

## Discussion

### Sequencing strategies and genome features

In the present study we sequenced and assembled the large ginkgo genome using high quality data. The genome (contig N50 of 48.2 kb and scaffold N50 of 1.36 Mb) is about 80 times larger than the *A. thaliana* genome, and is even larger than those of the angiosperm plants sorghum, maize and orchid that are renowned for their large genomes. The contiguity of the ginkgo genome is far better than previously published gymnosperm genomes, and may have benefited from several factors. Firstly, ginkgo DNA was extracted from the female gametophytic endosperm of a single seed based on the strategy described previously for the loblolly pine (*P. glauca*) genome [23], but *P. glauca* DNA was derived from apical shoots [52], which may have introduced considerable heterozygosity. Secondly, the amount of the sequencing data generated for ginkgo was much greater than that of other gymnosperms, resulting in a deeper coverage. We generated a total of ~1969 Gb raw data for ginkgo with a sequencing depth of 196.95, compared with 970 Gb raw data and 43.32-fold depth for *P. glauca* WS77111 [21]. Thirdly, we constructed three paired-end and five mate-pair libraries with sizes ranging from 250 to 40 kb, which facilitated sequence assembly, whereas for *P. glauca* WS77111 only three libraries were constructed, with fragment lengths of 400 bp, 600 bp and 15 kb. Moreover, the genomes of *P. glauca* and *P. taeda* are approximately twice the size of the ginkgo genome, and the repeat sequences in *P. taeda* amount to 79% of the total genome [21, 25]. Thus, we were able to generate a high quality assembly of the gymnosperm genome, which has long been presumed a huge challenge due to the genome size [53] and high proportion of repeat elements [54].

The ginkgo genome is mainly constituted by repetitive DNA sequences, with TEs accounting for at least 75%. This proportion is greater than reported for other sequenced plant genomes such as 70% in Norway spruce [19], 61% in orchid [55], 58% in sorghum [56], 49% in grape [57] and 35% in rice [58], but is still slightly smaller than the 79% in loblolly pine [23] (Additional file 1: Table S8). Based on our more complete and contiguous ginkgo genome, we constructed a repeat library that will likely prove more valuable for gymnosperm genome research than other currently available conifer genomes.

Our approach demonstrated the sequencing, assembly and annotation of a large genome, and provides insight into the *de novo* sequencing of other challenging genomes such as other gymnosperms and ferns [59]. Our approach may also facilitate improvements in the quality of gymnosperm genome data more generally.

### Evolution of gene structures and large genomes

Compared with the fern *S. moellendorffii*, the conifer *P. abies*, and the angiosperms *A. thaliana* and *O. sativa*, ginkgo genes are clearly much longer due to the insertion of TE. The length and number of exons among species is conserved, whereas the intron length varies dramatically among land plants. Like genes, introns in ginkgo are also much longer than in other land plants (Fig. 1c, Additional file 2: Figures S6 and S7). Across all gingko genes, 16,939 introns were longer than 20 kb, accounting for 12.45% of all introns, a significantly higher percentage than reported for conifers [40]. The average intron length was not correlated with genome size in conifers [40], and this was also supported by the ginkgo genome data in the present study. Although introns in Ginkgo are longer on average, the genome is only half the size of the *P. abies* genome (Additional file 2: Figure S7). These findings indicate a remarkable expansion of introns and a complex evolution of gene structure in the ginkgo genome.

The present results showed that the genome of ginkgo comprises a high proportion of repetitive sequences (at least 76.58%) with long introns with TE insertions. LTR elements appeared to have accumulated gradually over time, but a significant amplification and accumulation was evident between 16 and 24 mya (Fig. 2c), consistent with their proposed gradual accumulation and an overall gradual increase in genome size [19]. Compared with Norway spruce, the LTR-RTs superfamilies *Ty3/Gypsy* and *Ty1/Copia* substantially more diverse and abundant in ginkgo, indicating greater expansion and divergence in the wider ginkgo genome. It should be noted that this conclusion might be biased to some extent due to disparities in the volumes of ginkgo and spruce datasets (24,090 vs. 2416; 17,564 vs. 1790) as well as the continuity of assembled sequences. Ginkgo has a much larger genome and a comparable proportion of LTR-RTs, whereas maize appears to have undergone a recent massive expansion in a limited number of genes in both *Ty3/Gypsy* and *Ty1/Copia* superfamilies, but LTR-RTs likely expanded in multiple early-to-late-divergence events across many subfamilies in ginkgo (Fig. 2a and b).

Our evidence supports two WGDs in ginkgo, with an ancient WGD occurring 515 to 735 mya, consistent with previous reports [46–48, 60, 61], and a more recent event reported for the first time in this study and not yet observed in other seed plants [46]. This recent WGD may have uniquely contributed to the morphological and biological diversity of the gingko lineage. In summary, both LTR-RT insertions and two WGD events may have contributed to the large genome of ginkgo.

The recent proliferation in the maize genome may have occurred ~3 mya or even more recently [62, 63], and therefore postdated the recent WGD event that occurred between 5 and 12 mya [62]. In the maize genome, chromosomal breakages and fusions, as well as uneven gene loss, may have occurred when the ancestral cell returned to a genetically diploid state after a WGD event [62]. This active mechanism might prevent a continuous growth in genome size in angiosperms. Thus, the lack of an active mechanism for removing transposable elements such as unequal recombination [64] might have led to the enormous genome size in gymnosperms [19].

### Multiple defense mechanisms underlie resilience in gingko

Ginkgo is renowned for its wide spectrum of resistance and tolerance of insects and pathogens, due to multiple chemical defense mechanisms including repellence and anti-feedance towards insect herbivores exerted by terpenic lactones (ginkgolides and bilobalides) [12], chemical toxicity towards insects and pathogens via flavonoids and/or lactones, and attraction towards predators of herbivorous insects through terpene-composed VOCs [12, 13]. Interestingly, this impressive arsenal is shared by a number of plants, which begs the question of why these defense systems result in significantly less herbivore-mediated disease in ginkgo.

One explanation for the outstanding resistance displayed by gingko is a remarkable duplication of resistance genes and enrichment of relevant pathways. Gene duplication could increase the efficiency of resistance-related reactions through dosage effects, and might also provide a platform for adaptive evolution of duplicated copies [55]. We revealed most notable gene duplications in at least two gene families responsible for biosynthesis of flavonoids and terpenes in ginkgo, respectively. Specifically, glucosinolate biosynthesis and α-bisabolene synthase gene families, comprising 29 and 28 genes, respectively, were highly duplicated in the gingko genome. The latter gene family, which is absent in both *A. thaliana* and *O. sativa*, plays an important role in terpene-mediated defense in conifers [34]. Meanwhile, biosynthesis pathways of flavonoids and terpenoids were enriched across multiple duplicate gene families (four for flavonoids and six for terpenoids). Furthermore, by comparing with all current species in the KEGG database, five terpenoid biosynthesis genes appear to be notably expanded in ginkgo, and this enrichment is likely to further increase the efficiency of biosynthesis.

Another explanation is the enrichment of ginkgo-specific gene families and genes involved in the biosynthesis of defense metabolites. Gene families unique to ginkgo are concentrated in pathways for monoterpenoid and phenylpropanoid biosynthesis (Additional file 1: Table S10) and in pathways involving biotic stimuli responses, defense responses, and terpene synthase activity (Fig. 4a). These species-specific gene families might add extra dosage effects that enhance resistance and possibly also adaptability.

The induction of direct defense metabolites such as flavonoids and the release of VOCs have been reported for many angiosperms [13]. Given the long-standing interaction between plants and insects, these shared strategies may suggest an ancient origin for these defense mechanisms. This hypothesis is further supported by the observation that the fern *Pterisvittata* also responds to herbivory by reactive oxygen species production and the emission of VOCs [65].

In contrast to well-studied chemical defense mechanisms, very little is known about bacterial resistance and the genes responsible in ginkgo. In the present study, we identified one gene family (containing *FLS*2 and *EFR*) involved in recognizing bacterial infection in gingko [50]. A striking incidence of genes duplication in this family has resulted in 62 members, far exceeding the number in *A. thaliana*. Furthermore, in gingko, this pathway (map04626) is enriched with an additional five gene families (*RIN4*, *HSP*90, *CEBiP*, *CaMCML*, and *PR*1) in addition to *FLS*2 and *EFR*.

## Methods

### Sampling and extraction of DNA and RNA

Multiple seeds at visually different developmental stages were collected from five separate large ginkgo trees at one of the ginkgo refuge populations located on Tianmu Mountain [8, 9], Zhejiang Province, China on 19 July 2015. Voucher specimens were deposited at the Herbarium of Zhejiang University (Zhou WB & Zhao LJ, HZU13445, HZU13446, HZU13448, HZU13449, HZU13453, HZU13459). Endosperm tissues, which directly develop from female gametophytes without fertilization and thus contain haploid genomes, were dissected from the seeds. Genomic DNA was extracted using a standard cetyltrimethylammonium bromide (CTAB) protocol [66]. The DNA yield of each seed was quantified (Additional file 1: Table S4) to select the candidate sample(s) for *de novo* sequencing. DNA extracted from a single seed of one sampled tree (TM011) was determined for the subsequent construction of all paired-end libraries. Other seeds from the same tree were used to construct long-insert libraries.

For RNA isolation, ovulate (female) and staminate (male) strobili and seedlings were collected from Tianmu Mountain in April 2015. Female and male samples from three to four ginkgo trees were pooled as mixed female and male samples (GinkgoF vs. GinkgoM), respectively. All aerial parts from three 2-year seedlings were also pooled as a mixed sample (GinkgoL1). Mixed samples were immediately frozen in liquid nitrogen and stored at -80 °C. Total RNA was isolated using a modified CTAB protocol [67].

### Library construction and sequencing

Due to the large size of the gingko genome (approximately 10 Gb), we constructed libraries with a wide range of insert sizes, aiming to alleviate the technical challenge of genome assembly. Eight libraries were built with insert sizes between 250 and 40 kb according to the manufacturer’s instructions. A total of 1969.48 Gb of raw data was generated using Illumina Hiseq 2000/4000 (see details in Additional file 1: Table S1) and clean data totaled 1253.09 Gb (Additional file 1: Table S2).

For transcriptome sequencing, 20 μg RNA from each tissue was used for cDNA library preparation and sequencing as described previously [29]. RNA integrity was assessed using capillary electrophoresis on an Agilent BioAnalyzer 2100 (Agilent Technologies, Palo Alto, California, USA). Polyadenylated RNA isolated using oligo (dT)-attached beads was fragmented and reverse transcribed to cDNA. Paired-end libraries generated from each tissue were sequenced separately on an Illumina Hiseq 2000 platform. A total of 19.10 Gb raw data were obtained for three samples, specifically 6.40 Gb, 6.30 Gb and 6.40 Gb for male, female and seedling samples, respectively (Additional file 1: Table S3).

### Genome assembly

Raw genomic sequencing data were filtered using SOAP*filter* (V2.2). Low-quality reads including those with adapters, with a high N percentage, and low quality bases (more than 10% of bases with a Q-phred value lower than 7 and 15 for Hiseq 2000 and Hiseq 4000 platform, respectively), and PCR duplicated reads were removed. Genome size and complexity were estimated using k-mer analysis on approximate 50-fold sequencing reads from small insert size libraries. Filtered reads were processed to generate the assembled genome using SOAP*denovo2* [68] (V2.0.4) with key parameters of -R -K 89 -D 3 -d 3. Gap filling was performed based on the assembly results utilizing KGF [69] (V1.06) and Gapcloser [70] (V1.10).

RNA-seq data from seeds and leaves were downloaded from NCBI (SRR2163235 and SRR1722455, respectively) and assembled using Trinity (V2012-10-05) [71], resulting in the assembly of 42,243 and 32,088 transcripts, respectively. Gingko ESTs were retrieved from the NCBI EST database. Assembled transcripts and retrieved ESTs were mapped to the ginkgo genome using blat [72] (V34) with default parameters and the sim4 output format. The blat results were filtered with a cutoff of 0.90 for identity and coverage.

### Repeat annotation

TEs were found to account for a large proportion of DNA in conifer genomes [54]. TEs and other repeat elements in the ginkgo genome were identified using a combination of homology-based and *de novo* approaches. Specifically, TEs at DNA and protein levels were determined based on repeat searching in the known repeat database Repbase [73] using RepeatMasker (V4.0.5) and RepeatProteinMask (V4.0.5). *De novo* repeat libraries were constructed based on the genome using the *de novo* prediction programs RepeatModeler (V1.0.8) and LTR-FINDER (V1.0.6), followed by the removal of contamination and multi-copy genes from libraries. Using these libraries as a database, repeats in the genome were identified and classified using RepeatMasker. Tandem repeats including microsatellites (SSRs) with unit sizes ranging from 1 to 2000 bases were identified using the program Tandem Repeats Finder (TRF, V4.07). Finally, non-redundant repeat content was calculated and summarized using an in-house Perl script (included in GigaDB [30]).

Intact LTR elements were detected using LTR_STRUC with default settings. Since loose default settings could lead to false positive results, results should be interpreted with caution. Pairs of LTR sequences were extracted and aligned using MUSCLE [74] (V3.8.31). The nucleotide distance *K* between one pair of LTRs was calculated using the Kimura two-parameter model in the distmat program within the EMBOSS package. Finally, the insert time *T* was calculated based on the formula *T* = *K* / 2*r* with a mutation rate (*r*) of 2.2 × 10^-9^ substitutions per base per year [45].

### Identification of LTR-RT elements

Two-round searches to identify *Ty1/Copia* and *Ty3/Gypsy* superfamilies in the genome of ginkgo and the other four land plants (*P. patens*, *P. abies*, *P. trichocarpa* and *Z. mays*) were performed based on the reported *Ty3/Gypsy* and *Ty1/Copia* reverse transcriptase domains with sequences EAYLDDLASRSRKRKDHPTHLRLIFERCRYFRIRLNPNKCSFCVTSGRLLGFIVSTTGIMVDPLKVGAIVQLPPPRTIVQLQSLQGKANFLRRFIANYAE and WKVYQMDVKSAFLNGYLEEEVYVQQPPRYEVRGQEDKVYRLKKALNGLKQAPRAWYSKIDSYMIKNEFIRSTSEPTLYTKVNEQGQILIVCLYVDDLIY, respectively. Genome sequences of Norway spruce were downloaded from database [75]. The method was as follows:

1. We searched the *Ty1-* and *Ty3-*specific domain amino acid sequences using tBLASTn (V2.2.26) against whole genome sequences for gingko and the other four species using parameters -p tBLASTn -e 1e-5 -F F -m 8. Target hits were obtained using a strict filter criteria of identity ≥0.50 and coverage ratio ≥0.95”.
2. Sequence hits obtained from round one searches were used as queries for the second round of tBLASTn searches for all genome sequences using an enhanced filter criteria of identity ≥0.80 and coverage ratio ≥0.95. The resulting nucleotide sequences were re-aligned against the amino acid sequences of the two domains using tBLASTn with parameter -m 0, leading to the eventual identification of amino acid sequences for all *Ty1* and *Ty3* elements.

To construct *Ty1* and *Ty3* phylogenetic trees for the five species (*G. biloba*, *P. patens, P. abies, P. trichocarpa* and *Z. mays*), the resultant amino acid sequences were aligned using MUSCLE [74] (V3.8.31) with default parameters. Phylogenetic trees were inferred based on multiple sequence alignment using FastTree [76] (V2.1.9) with parameters of amino acid distances: BLOSUM45, joins: balanced, support: SH-like 1000, search: normal + NNI + SPR (2 rounds range 10) + ML-NNI, opt-each = 1, tophits: 1.00*sqrtN, close = default refresh = 0.80, ML model: Jones-Taylor-Thorton, and CAT approximation with 20 rate categories.

### Gene model annotation

Protein sequences of *S. moellendorffii*, *P. abies*, *P. taeda*, *A. thaliana* and *O. sativa* were aligned against the ginkgo genome using tBLASTn (V2.2.26). Gene structure was predicted using GeneWise [77] (V2.2.0). Transcripts from seeds and leaves assembled from gingko RNA-seq and EST data separated from EST data in NCBI were mapped to the genome. The program PASA [78] was applied to assemble the spliced alignment results and annotate the candidate genes. Genes were predicted using the hidden Markov model in Augustus (V3.0.3) through a *de novo* approach. Results derived from different methods were integrated, and only those candidate genes consistently supported were retained in the final gene set. For function annotation, protein sequences were mapped to the Gene Ontology [79], KEGG [80], InterPro [81], UniProt [82] and Non-redundant protein NCBI databases.

### Detection of tandem duplicate genes

Pairwise self-alignment of protein sequences was conducted using BLASTp (V2.2.26), and protein sequences were sorted by coordinates in the assembled sequences. Genes with a distance greater than 2 were filtered, where the gene distance represents the number of genes between two focal genes. TDGs were calculated for *G. biloba*, *Z. mays*, *A. thaliana*, *P. trichocarpa* and *G. max*.

### Analysis of phylogenetic reconstruction and evolution

CDSs of *S. moellendorffii*, *G. biloba*, *A. thaliana* and *O. sativa* were extracted based on genome annotation. Transcriptome sequencing data for *P. canatriensis* and *C. revoluta* were downloaded from NCBI (SRR1531151 and SRR1525778), and RNA-seq data were assembled using Trinity [71], resulting in 78,805 and 68,553 transcripts, respectively. The EMBOSS sixpack package [83] was employed to translate all possible proteins and to filter candidates by mapping to the NCBI plant protein database. A total of 28,925 and 32,512 proteins were annotated based on the transcriptomes of *P. canatriensis* and *C. revoluta*, respectively*.* OrthoMCL (version 5) [44] was used to cluster CDS from all six species and to identify the gene families. In total, 920 single copy genes were identified, and their phase1 sites were used to reconstruct phylogenetic trees using PhyML (V3.0) [84]. To investigate the expansion and contraction of gene families, changes were identified by comparing 7795 gene families filtered from all 24,271 gene families using CAFÉ [85].

### Identification of WGD events

All gingko amino acid sequences were self-aligned using BLASTp with filtering criteria identity ≥0.40, e value ≤1.0 × 10^-5^, more than 100 amino acids matched. To obtain paralogous gene families, we performed gene cluster analyses based on the CDS alignment. *K*_s_ values were calculated for each paralogous family using yn00 in the PAML package. The *K*_s_ of a given family was represented by the median value, and the distribution of corrected *K*_s_ values was plotted by masking TDGs.

## Additional files

Additional file 1: Table S1. Statistics for gingko genome raw sequencing data. **Table S2.** Statistics for ginkgo genome clean sequencing data. **Table S3.** Statistics for RNA-seq data from male and female ginkgo samples. **Table S4.** Comparisons of DNA yield and genome survey analysis. **Table S5.** Transposable elements in the ginkgo genome. **Table S6.** Gene model annotation. **Table S7.** Gene functional annotation. **Table S8.** Comparison of gene metrics for the genomes of ginkgo and seven other land plants. **Table S9.** Comparison of gene family clusters between ginkgo and five other land plants. **Table S10.** Functional enrichment of specific genes in ginkgo. **Table S11.** Comparison of TDGs in the genomes of ginkgo and seven other land plants. (DOCX 41 kb)
Additional file 2: Figure S1. 17-mer frequency distribution of ginkgo genome sequencing. **Figure S2.** Distribution of sequence depth for the ginkgo genome. The x-axis represents the depth and the y-axis represents the proportion of the corresponding DNA bases. Clean reads were aligned against genic regions and the percentage of bases with different sequencing depth was calculated. **Figure S3.** The distribution of sequence divergence rates of TEs in the ginkgo genome. (a) Based on the Repbase-comparison approach; (b) Based on the de novo approach. **Figure S4.** Comparison of the length distribution of gene sets in ginkgo and four other land plants. **Figure S5.** Comparison of orthologous genes in ginkgo and five other land plants. **Figure S6.** Comparison of gene structure in G. biloba and four other land plants. Five land plants are included in each gene cluster: A. thalina in orange, G. biloba in yellow, O. sativa in green, P. abies in pink and S. moellendorffii in blue. **Figure S7.** Comparison of genome size and average intron length between ginkgo and four other land plants. Note that the intron size in P. abies might be underestimated due to the assembly quality of its genome. **Figure S8.** Comparison of the distribution of repeat elements between intergenic and intron regions in ginkgo. (DOCX 641 kb)

## Abbreviations

CDS: Coding sequence
EFR: EF-Tu receptor
ESTs: Expressed sequence tags
FLS: Flavonol synthase
FLS2: Flagellin-sensitive 2
LRR: Leucine rich repeat
LTR-RTs: Long terminal repeat retrotransposons
mya: Million years ago
TDG: Tandem duplicate gene
TE: Transposable element
VOC: Volatile organic compound
WGD: Whole genome duplication
