# Supplementary material for: Draft genome of the living fossil Ginkgo biloba
Source: Gigascience. 2016 Nov 21;5:49. doi: 10.1186/s13742-016-0154-1 (PMC5118899; doi:10.1186/s13742-016-0154-1)
Supplement: Additional file 1: Table S1. — Statistics for gingko genome raw sequencing data. Table S2. Statistics for ginkgo genome clean sequencing data. Table S3. Statistics for RNA-seq data from male and female ginkgo samples. Table S4. Comparisons of DNA yield and genome survey analysis. Table S5. Transposable elements in the ginkgo genome. Table S6. Gene model annotation. Table S7. Gene functional annotation. Table S8. Comparison of gene metrics for the genomes of ginkgo and seven other land plants. Table S9. Comparison of gene family clusters between ginkgo and five other land plants. Table S10. Functional enrichment of specific genes in ginkgo. Table S11. Comparison of TDGs in the genomes of ginkgo and seven other land plants. (DOCX 41 kb) [file 13742_2016_154_MOESM1_ESM.docx]

**Supplementary Table 1. Statistics for gingko genome raw sequencing data**

| **Paired-end**  **libraries** | **Insert size** | **Average read**  **length (bp)** | **Total data (Gb)** | **Sequence**  **depth (X)** |
| --- | --- | --- | --- | --- |
| Illumina reads | 250 bp | 150 | 1080.73 | 108.07 |
|  | 500 bp | 100 | 132.71 | 13.27 |
|  | 800 bp | 100 | 42.39 | 4.24 |
|  | 2 kb | 75 | 205.09 | 20.51 |
|  | 5 kb | 75 | 109.91 | 10.99 |
|  | 10 kb | 75 | 113.84 | 11.38 |
|  | 20 kb | 75 | 207.51 | 20.75 |
|  | 40 kb | 100 | 77.30 | 7.73 |
| Total | ---- | -- | 1969.48 | 196.95 |
| The genome size (10.00 Gb) was estimated using k-mer analysis on sample TM011. | | | | |

**Supplementary Table2. Statistics for ginkgo genome clean sequencing data**

| **Pair-end libraries** | **Insert size** | **Average read length (bp)** | **Total data (Gb)** | **Sequence**  **depth (X)** |
| --- | --- | --- | --- | --- |
| Illumina reads | 250 bp | 140 | 906.70 | 90.67 |
|  | 500 bp | 90 | 108.95 | 10.89 |
|  | 800 bp | 90 | 35.23 | 3.52 |
|  | 2 kb | 70 | 97.88 | 9.79 |
|  | 5 kb | 70 | 35.37 | 3.54 |
|  | 10 kb | 70 | 31.90 | 3.19 |
|  | 20 kb | 70 | 27.85 | 2.78 |
|  | 40 kb | 80 | 9.22 | 0.92 |
| Total | ---- | -- | 1253.09 | 125.31 |
| The genome size (10.00 Gb) was estimated using k-mer analysis on sample TM011. | | | | |

**Supplementary Table 3. Statistics for RNA-seq data from male and female ginkgo samples**

| Sample | Total data^*^ (Gb) | Read length (bp) | Number of reads | Q20^**^ of reads1 (%) | Q20 of reads2 (%) | | Q30^***^ of reads1 (%) | | Q30 of reads2 (%) |
| --- | --- | --- | --- | --- | --- | --- | --- | --- | --- |
| Male | 6.40 | 125 | 53,768,792 | 98.41 | | 95.88 | | 95.81 | 91.99 |
| Female | 6.30 | 125 | 52,936,244 | 98.34 | | 95.38 | | 95.62 | 91.17 |
| Seedling | 6.40 | 125 | 53,768,792 | 98.80 | | 97.74 | | 96.67 | 94.69 |

* Total size of sequenced raw data. All other column descriptions were obtained from clean data

**Base with quality value of 20 or higher

*** Base with quality value of 30 or higher

**Supplementary Table 4. Comparisons of DNA yield and genome survey analysis**

| **Sample name** | **Total DNA (μg)** | **Sequence depth** | **Survey genome size** | **Assembly N50 results** |
| --- | --- | --- | --- | --- |
| TM010 | 39.03 | 22.80 | 9,045,858,698 | 13,878/1,192 |
| TM011 | 28.64 | 23.82 | 10,002,180,011^*^ | 15,886/1,434 |
| TM2217 | 32.93 | 23.90 | 9,252,852,345 | 17,150/1,516 |

*The genome size was estimated using k-mer analysis

**Supplementary Table 5. Transposable elements in the ginkgo genome**

|  | **RepBase TEs** | | **TE proteins** | | ***De novo*** | | **Combined TEs** | |
| --- | --- | --- | --- | --- | --- | --- | --- | --- |
|  | **Length (bp)** | **% in genome** | **Length (bp)** | **% in genome** | **Length (bp)** | **% in genome** | **Length (bp)** | **% in genome** |
| **DNA** | 91,123,939 | 0.859 | 43,355,673 | 0.409 | 314,325,967 | 2.963 | 354,935,994 | 3.346 |
| **LINE** | 57,042,880 | 0.538 | 184,605,961 | 1.740 | 376,759,434 | 3.551 | 460,463,526 | 4.340 |
| **SINE** | 77,400 | 0.001 | 0 | 0.000 | 184,380 | 0.002 | 261,728 | 0.002 |
| **LTR** | 1,230,790,102 | 11.602 | 2,518,147,103 | 23.737 | 6,337,117,496 | 59.735 | 6,434,519,114 | 60.653 |
| **Other** | 452 | 0.000 | 0 | 0.000 | 0 | 0.000 | 452 | 0.000 |
| **Unknown** | 0 | 0.000 | 0 | 0.000 | 2,694,184,164 | 25.396 | 2,694,184,164 | 25.396 |
| **Total** | 1,338,920,079 | 12.621 | 2,739,522,408 | 25.823 | 7,906,768,698 | 74.531 | 7,992,693,151 | 75.341 |

**Supplementary Table 6. Gene model annotation**

| **Gene set** | | **Number** | **Average transcript length (bp)** | **Average CDS length (bp)** | **Average exon per gene** | **Average exon length (bp)** | **Average intron length (bp)** |
| --- | --- | --- | --- | --- | --- | --- | --- |
| ***De novo*** | AUGUSTUS | 132,628 | 1495 | 735 | 3.45 | 213 | 310 |
| **Homolog** | *S. moellendorffii* | 36,198 | 7619 | 671 | 2.38 | 281 | 5018 |
|  | *P. abies* | 57,372 | 3963 | 660 | 2.20 | 300 | 2748 |
|  | *P. taeda* | 42,971 | 555 | 322 | 1.32 | 244 | 725 |
|  | *A. thaliana* | 34,124 | 10,406 | 833 | 2.84 | 294 | 5216 |
|  | *O. sativa* | 18,481 | 8291 | 1069 | 3.05 | 351 | 3528 |
| **EST** | | 20,932 | 22,961 | 596 | 2.23 | 267 | 18,220 |
| **EST-seed** | | 40,207 | 43,578 | 1347 | 3.50 | 385 | 16,908 |
| **EST-leaf** | | 41,543 | 40,849 | 1268 | 3.24 | 392 | 17,679 |
| **RNA-Seq** | | 45,194 | 42,358 | 1684 | 4.48 | 376 | 11,688 |
| **Final set** | | 41,840 | 26,829 | 1186 | 4.25 | 279 | 7884 |

**Supplementary Table 7. Gene functional annotation**

|  | | **Number** | **Percent (%)** |
| --- | --- | --- | --- |
| **Total** | | 41,840 |  |
| **Annotated** | InterPro | 21,989 | 52.55 |
|  | GO | 15,680 | 37.48 |
|  | KEGG | 21,542 | 51.49 |
|  | Swissprot | 21,763 | 52.01 |
|  | TrEMBL | 28,158 | 67.30 |
| **Unannotated** | | 13,340 | 31.88 |

**Supplementary Table 8. Comparison of gene metrics for the genomes of ginkgo and seven other land plants**

|  | Number of genes | Genome size (Mb) | TE content (%) | Average intron length (bp) |
| --- | --- | --- | --- | --- |
| *Ginkgo biloba* | 41,840 | 10,609 | 75 | 7884 |
| *Pinus taeda* | 50,172 | 20,148 | 79 | 2741 |
| *Picea abies* | 58,587 | 19,000 | 70 | 1020 |
| *Picea glauca* | 16,386 | 22,390 | NA | 3900 |
| *Phalaenopsis equestris* | 29,431 | 1086 | 61 | 2922 |
| *Sorghum bicolor* | 27,159 | 738 | 58 | 436 |
| *Vitis vinifera* | 25,329 | 486 | 49 | 968 |
| *Oryza sativa* | 37,544 | 388 | 35 | 413 |

**Supplementary Table 9. Comparison of gene family clusters between ginkgo and five other land plants**

| **Species** | **# Total genes** | **# Unclustered genes** | **# Genes in families** | **# families** | **# Unique families** | **# Average genes per family** |
| --- | --- | --- | --- | --- | --- | --- |
| *G.biloba* | 41,840 | 26,330 | 15,510 | 12,303 | 2018 | 2.14 |
| *P. canariensis* | 28,858 | 23,808 | 5050 | 11,153 | 1464 | 2.13 |
| *S. moellendorffii* | 22,212 | 17,244 | 4968 | 9454 | 1659 | 1.82 |
| *C. revoluta* | 32,471 | 26,695 | 5776 | 11,434 | 1922 | 2.33 |
| *O. sativa* | 38,942 | 27,411 | 11,531 | 13,043 | 2434 | 2.10 |
| *A. thaliana* | 26,637 | 22,333 | 4304 | 11,985 | 1261 | 1.86 |

**Supplementary Table 10. Functional enrichment of specific genes in ginkgo**

| **Pathway** | **Specific genes** | **All genes** | ***P* value** | **Pathway ID** |
| --- | --- | --- | --- | --- |
| ABC transporters | 451 (10.62%) | 605 (2.81%) | 6.43×10^-193^ | ko02010 |
| DNA replication | 59 (1.39%) | 143 (0.66%) | 2.80×10^-09^ | ko03030 |
| Monoterpenoid biosynthesis | 10 (0.24%) | 14 (0.07%) | 4.06×10^-05^ | ko00902 |
| Ribosome biogenesis in eukaryotes | 65 (1.53%) | 222 (1.03%) | 4.02×10^-04^ | ko03008 |
| Cyanoamino acid metabolism | 33 (0.78%) | 96 (0.45%) | 5.38×10^-04^ | ko00460 |
| Peroxisome | 71 (1.67%) | 258 (1.20%) | 1.49×10^-03^ | ko04146 |
| Phenylpropanoid biosynthesis | 91 (2.14%) | 347 (1.61%) | 1.86×10^-03^ | ko00940 |
| Diterpenoid biosynthesis | 27 (0.64%) | 80 (0.37%) | 2.24×10^-03^ | ko00904 |

**Supplementary Table 11. Comparison of TDGs in the genomes of ginkgo and seven other land plants**

| Species | Total genes | Tandem clusters ≥2 | Gene number | Tandem cluster ≥3 | Gene number | Tandem cluster ≥4 | Gene number |
| --- | --- | --- | --- | --- | --- | --- | --- |
| *G. biloba* | 41,840 | 2061 | 5201 | 614 | 2307 | 245 | 1200 |
| *P. abies* | 24,695 | 423 | 874 | 26 | 80 | 2 | 8 |
| *G. max* | 53,434 | 2819 | 7696 | 911 | 3880 | 414 | 2389 |
| *P. trichocarpa* | 40,996 | 2607 | 7657 | 948 | 4339 | 524 | 3067 |
| *Z. mays* | 39,893 | 1706 | 4227 | 422 | 1659 | 180 | 933 |
| *O. sativa* | 38,942 | 2259 | 6402 | 817 | 3518 | 406 | 2285 |
| *A. thaliana* | 26,637 | 1702 | 4598 | 541 | 2276 | 256 | 1421 |
| *V. vinifera* | 26,346 | 1699 | 5108 | 671 | 3052 | 361 | 2122 |
