# Supplementary material for: Draft genome of the living fossil Ginkgo biloba
Source: Gigascience. 2016 Nov 21;5:49. doi: 10.1186/s13742-016-0154-1 (PMC5118899; doi:10.1186/s13742-016-0154-1)
Supplement: Additional file 2: Figure S1. — 17-mer frequency distribution of ginkgo genome sequencing. Figure S2. Distribution of sequence depth for the ginkgo genome. The x-axis represents the depth and the y-axis represents the proportion of the corresponding DNA bases. Clean reads were aligned against genic regions and the percentage of bases with different sequencing depth was calculated. Figure S3. The distribution of sequence divergence rates of TEs in the ginkgo genome. (a) Based on the Repbase-comparison approach; (b) Based on the de novo approach. Figure S4. Comparison of the length distribution of gene sets in ginkgo and four other land plants. Figure S5. Comparison of orthologous genes in ginkgo and five other land plants. Figure S6. Comparison of gene structure in G. biloba and four other land plants. Five land plants are included in each gene cluster: A. thalina in orange, G. biloba in yellow, O. sativa in green, P. abies in pink and S. moellendorffii in blue. Figure S7. Comparison of genome size and average intron length between ginkgo and four other land plants. Note that the intron size in P. abies might be underestimated due to the assembly quality of its genome. Figure S8. Comparison of the distribution of repeat elements between intergenic and intron regions in ginkgo. (DOCX 641 kb) [file 13742_2016_154_MOESM2_ESM.docx]

**Supplementary Fig. 1 17-mer frequency distribution of ginkgo genome sequencing.**

**
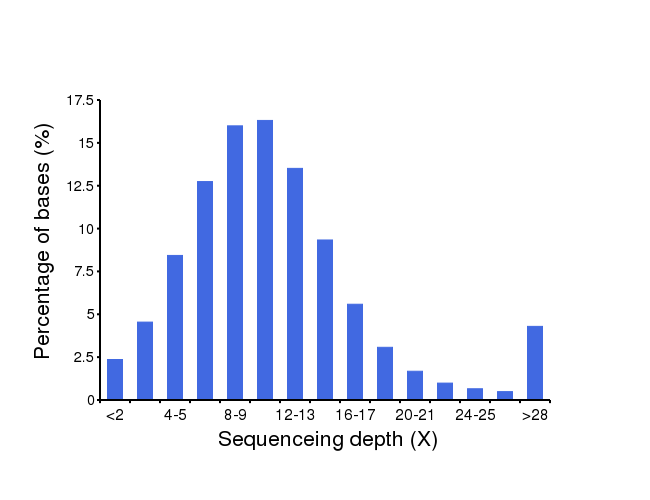
**

**Supplementary Fig. 2 Distribution of sequence depth for the ginkgo genome.** The x-axis represents the depth and the y-axis represents the proportion of the corresponding DNA bases. Clean reads were aligned against genic regions and the percentage of bases with different sequencing depth was calculated.


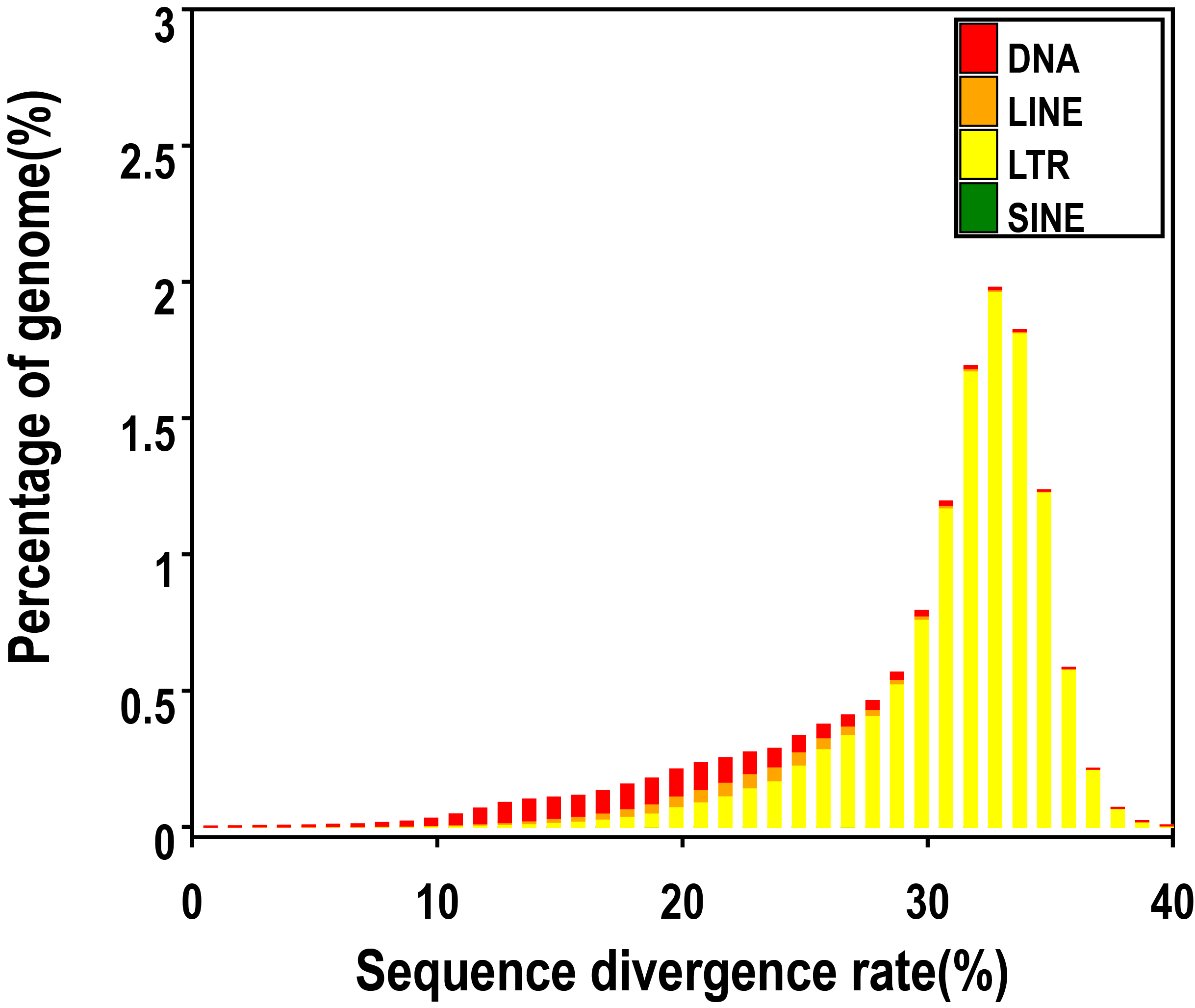

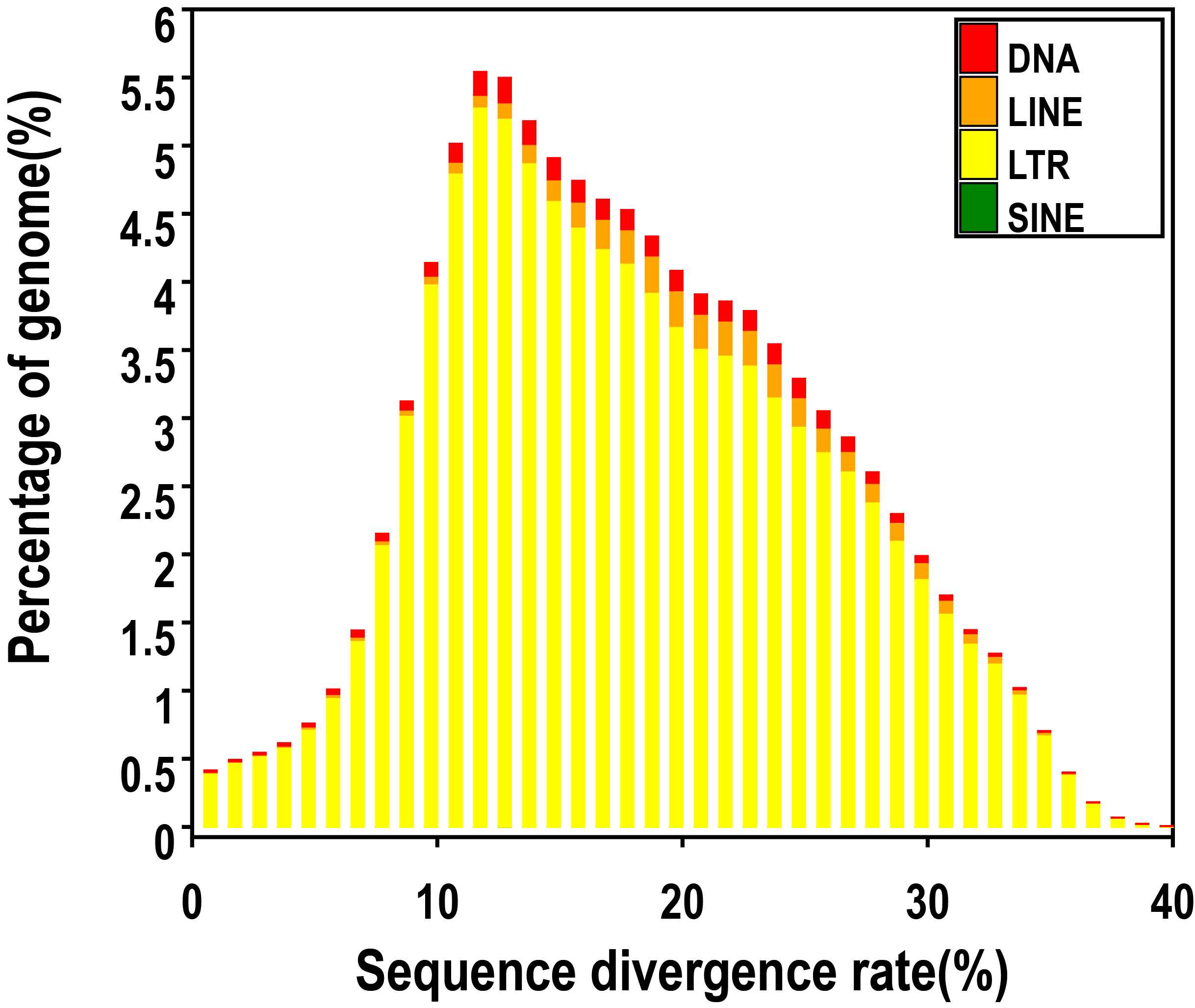


**a**

**b**

**Supplementary Fig. 3 The distribution of sequence divergence rates of TEs in the ginkgo genome. (a)** Based on the Repbase-comparison approach; **(b)** Based on the *de novo* approach.

**
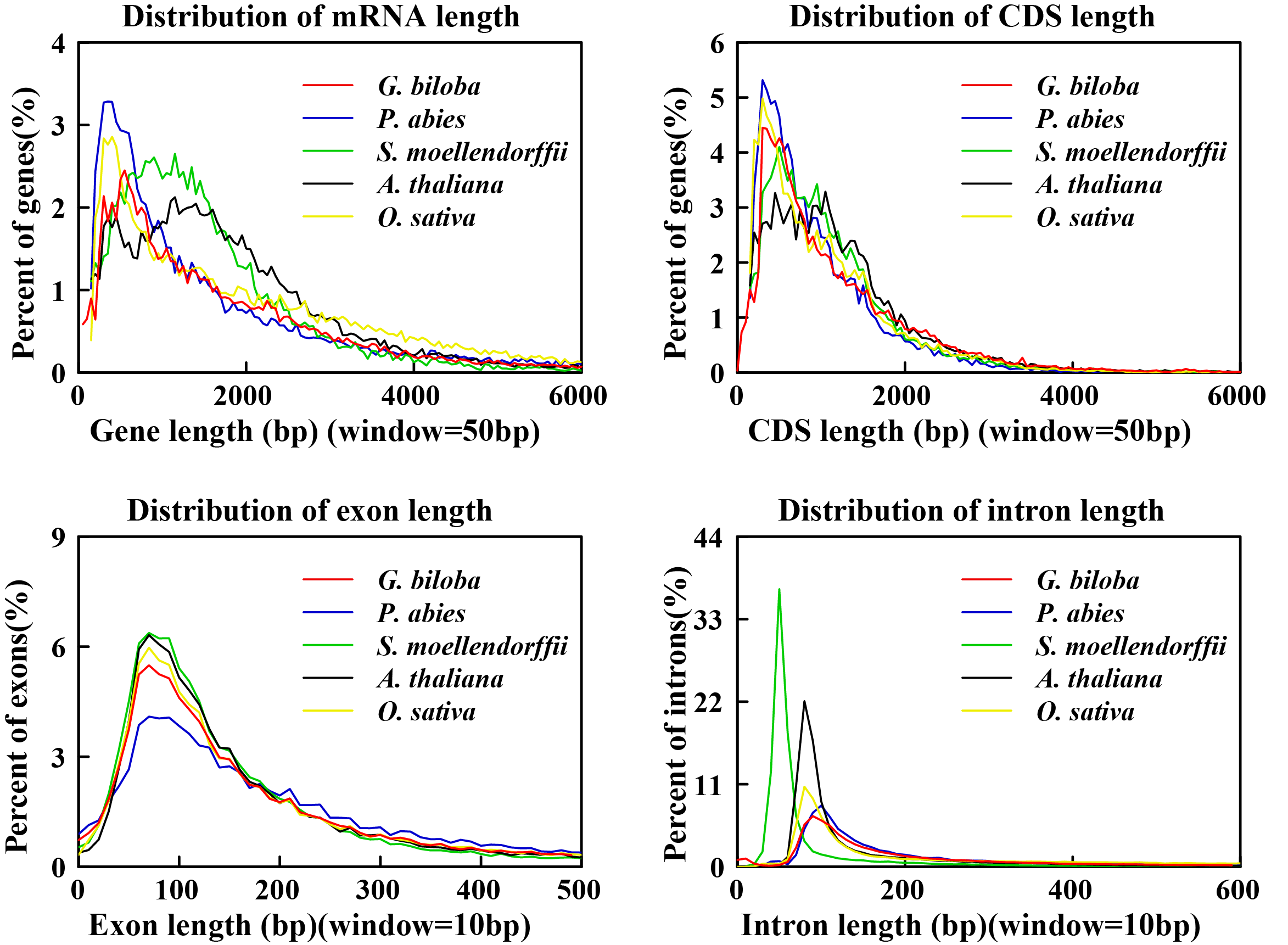
**

**Supplementary Fig. 4Comparison of the length distribution of gene sets in ginkgo and four other land plants.**


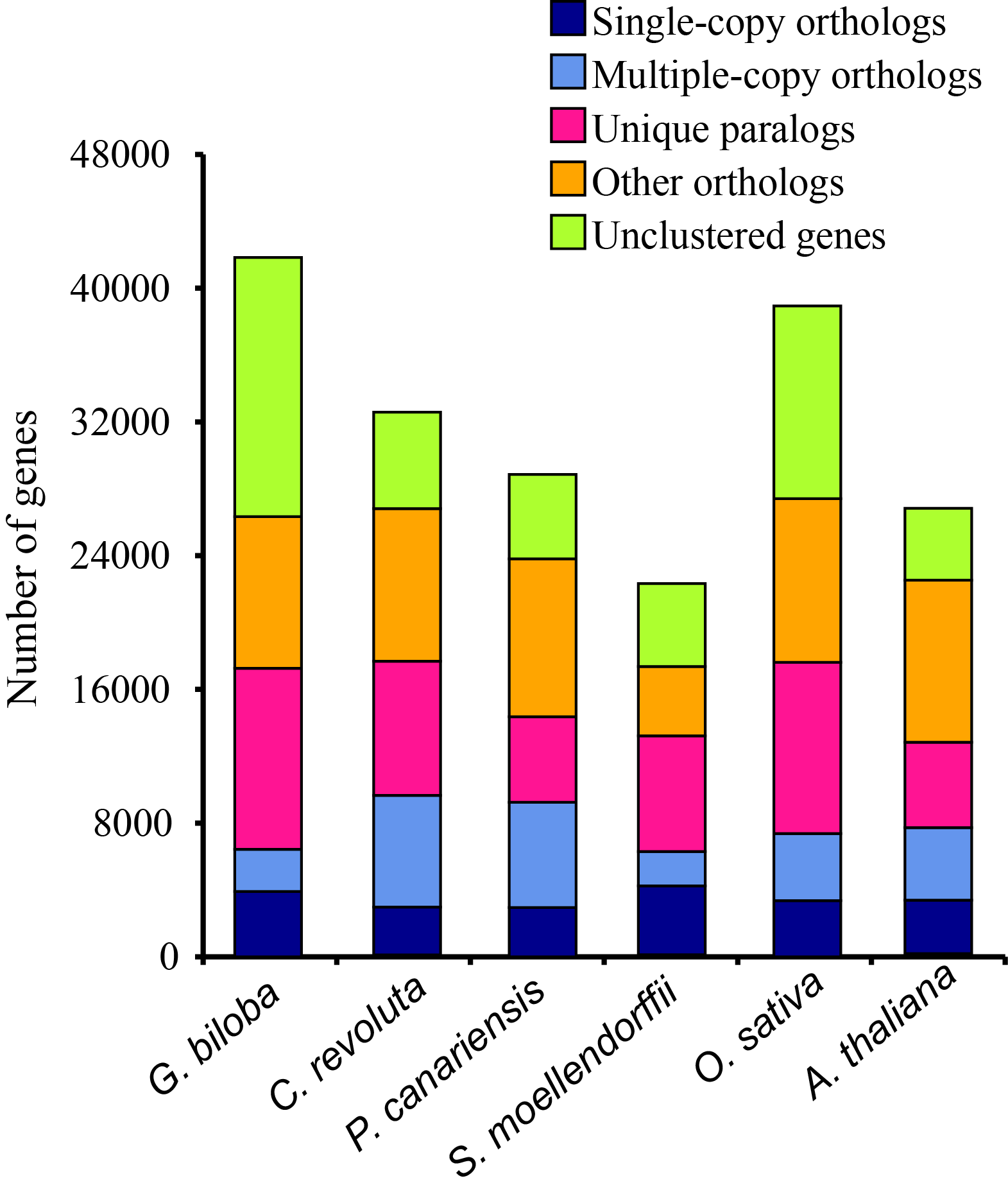


**Supplementary Fig. 5 Comparison of orthologous genes in ginkgo and five other land plants*.***


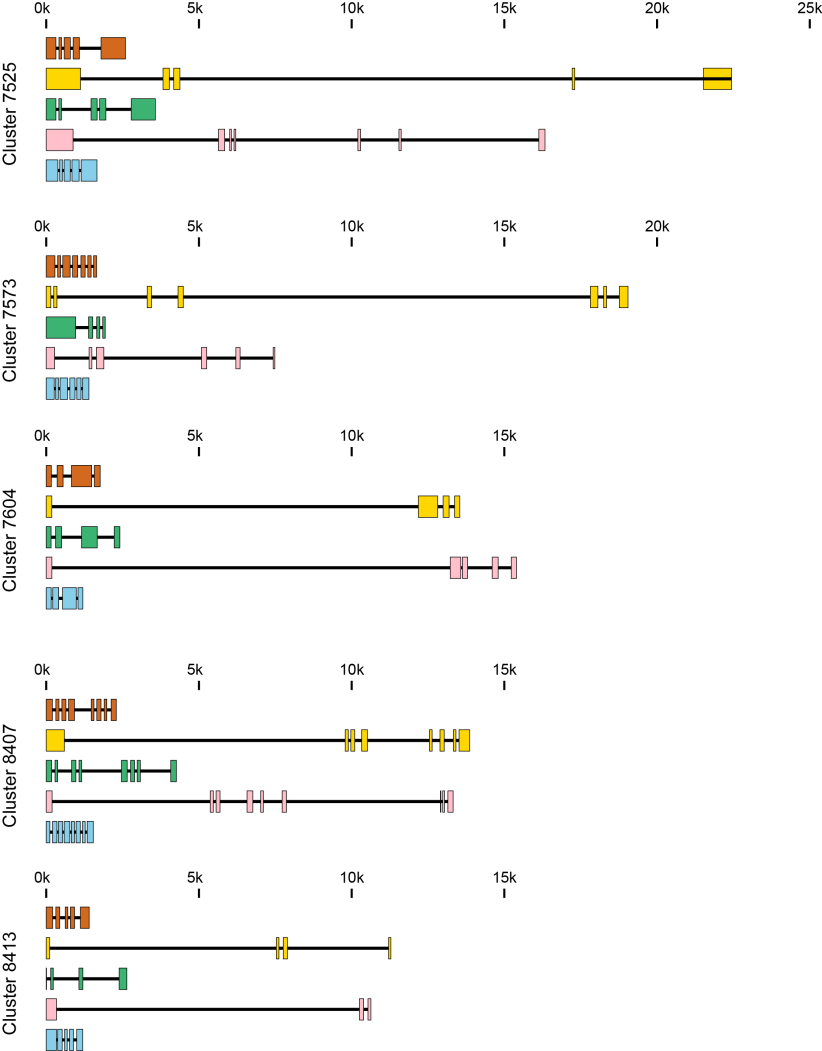


**Supplementary Fig. 6 Comparison of gene structure in *G. biloba* and four other land plants.** Five land plants are included in each gene cluster: *A. thalina* in orange*, G. biloba* in yellow*, O. sativa* in green*, P. abies* in pink and *S. moellendorffii* in blue.


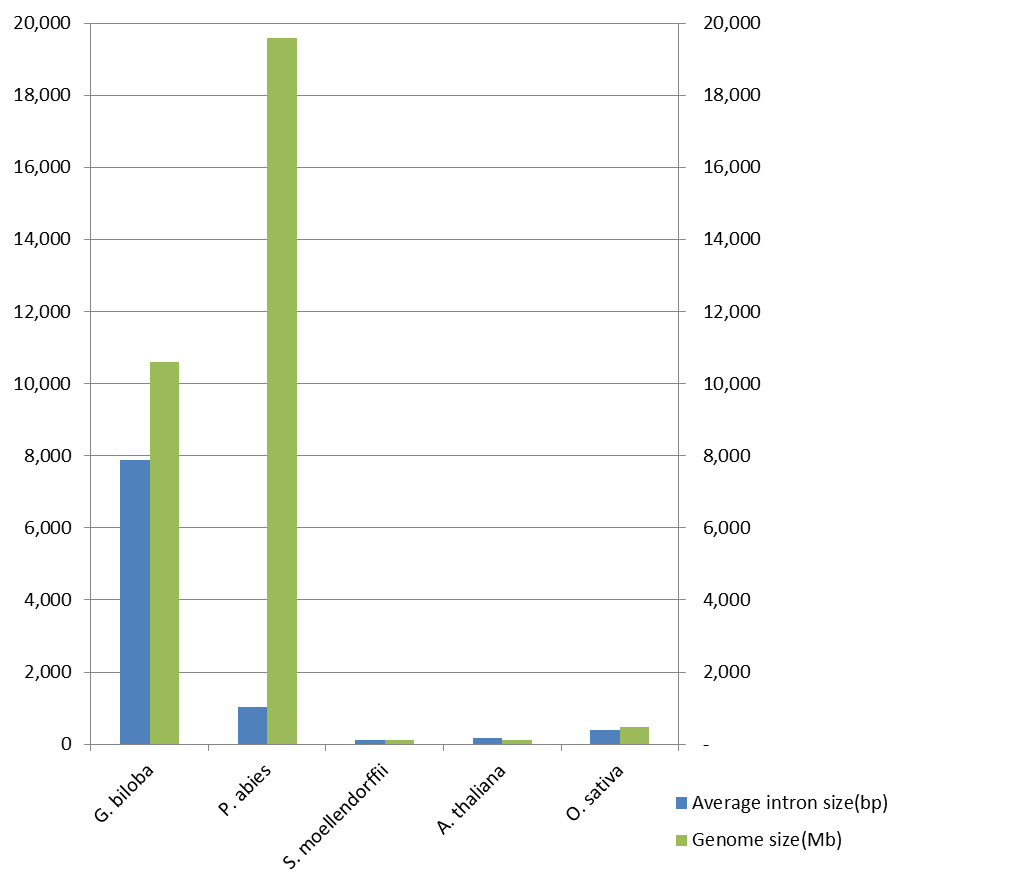


**Supplementary Fig. 7 Comparison of genome size and average intron length between ginkgo and four other land plants.** Note that the intron size in *P. abies* might be underestimated due to the assembly quality of its genome.

**Supplementary Fig. 8 Comparison of the distribution of repeat elements between intergenic and intron regions in ginkgo.**
